# Exposure to Nail and False Eyelash Glue: A Case Series Study

**DOI:** 10.3390/ijerph17124283

**Published:** 2020-06-15

**Authors:** Elena Brambilla, Marta Crevani, Valeria M. Petrolini, Giulia Scaravaggi, Maria Di Primo, Elisa Roda, Carlo A. Locatelli

**Affiliations:** 1 Pavia Poison Centre, National Toxicology Information Centre, Laboratory of Clinical & Experimental Toxicology, Toxicology Unit, Istituti Clinici Scientifici Maugeri IRCCS, 27100 Pavia, Italy; marta.crevani@icsmaugeri.it (M.C.); valeria.petrolini@icsmaugeri.it (V.M.P.); giulia.scaravaggi@icsmaugeri.it (G.S.); maria.diprimo@icsmaugeri.it (M.D.P.); elisa.roda@icsmaugeri.it (E.R.); carlo.locatelli@icsmaugeri.it (C.A.L.); 2 Department of Biology and Biotechnology “L. Spallanzani”, Laboratory of Cell Biology and Neurobiology, University of Pavia, 27100 Pavia, Italy

**Keywords:** nail glue, false eyelash glue, dermal/ocular, cyanoacrylate, chemical burns, accidental injury, epidemiology

## Abstract

The use of artificial nail tips in professional manicure services and the application of false eyelashes are a growing trend among young women. Often, this “beauty routine” is performed at home without the supervision of an expert beautician, raising health problems due to either the spillage of these products or to accidental exposure to children. The aim of this study is to review the Pavia Poison Control Centre clinical records to identify the frequency, the most common route of exposure, and the possible risks associated to these events to support the decision-making process in emergency departments. The Pavia Poison Control Centre database was retrospectively searched for records reporting nail or false eyelash glue exposure from January 2007 to April 2020, and 42 patients were identified. Among the patients, 76% presented symptoms from mild to severe, while 24% were asymptomatic. The most common route of exposure was dermal, through cutaneous contact, as determined for 19 patients involved. Among these, seven patients presented with second-degree chemical burns, cutaneous erythema, and ocular symptoms. The most dangerous glue component was cyanoacrylate, leading to symptoms in 16 out of 22 patients, while three cases remained asymptomatic. Even if this exposure is relatively rare, nail and false eyelash glue can be seriously harmful, especially when exposure occurs via dermal or ocular routes. In the case of emergency, it is important to treat the patient as fast as possible to limit the damage caused by a burn. Moreover, even though these products are often perceived as harmless, safety precautions should be taken to prevent children from accidental contact.

## 1. Introduction

Cosmetics are very popular products, and their use is continuously increasing, since caring about one’s appearance has become important; moreover, products can be bought online and the beauty procedure performed at home to limit costs. As a part of routine body care activity, the word “cosmetic” includes a huge number of different products, all regulated by The European Cosmetics Directive No. 1223/2009. As a general rule, a cosmetic product made available on the European market should be safe for human health when used under normal or reasonably foreseeable conditions of use [1,2].

More than one cosmetic product can be found in everyone’s homes, so growing concerns come from the possible harmful effects of cosmetic beauty products on human health, particularly in cases of misuse, or accidental contact with children [3,4].

In the last 10 years, the cosmetic trend of artificial nails has gained popularity, especially in developed countries. It has become the semi-permanent alterative to the simple manicure. Acrylic nails are a highly popular beauty trend due to their longevity and unique and eye-catching designs. This beauty process should be conducted by a nail technician, as there are many skills and techniques only a professional can achieve. Nonetheless, based on the frequent need to re-fill these nails, with the aim at reducing expensive costs, this beauty routine is often performed at home, without the supervision of an expert beautician. This unwise home or self use of nail or false eyelash glue can raise health problems due to the spillage of this adhesive or to accidental child exposure.

Concerning nail glue, also sold as false eyelash glue, its composition is mainly based on a mixture of alcohol, cyanoacrylate, or photo-bonded methacrylate [5]. When the pre-polymer composed of formaldehyde and alkyl cyanoacetate is depolymerized, the liquid monomer of cyanoacrylate is obtained [6]. In the presence of water or in contact with hydroxyl groups (-OH), cyanoacrylate rapidly polymerizes, forming long and strong chains responsible for bonding; its side chains are also responsible for its property and different uses, and indeed, many different forms of cyanoacrylate exist as medical-grade glues used for oculoplastics, skin closure, and other surgical procedures. The longer the chain compounds, the slower it degrades, with lower reactivity and toxicity [6]. Commercial cyanoacrylates used as “fast-acting” adhesives for DIY works are more irritating to skin tissue and have higher toxicity [7,8,9].

In the literature, the main risk reported for acrylic and artificial nails is allergies, and, indeed, allergic and irritant reactions, such as dermatitis, may develop in frequent acrylic nail bearers or in beauticians and professionals [10,11].

A case series of exposure to nail/false eyelash glue is here reported, with the aim of identifying the frequency, the most common route of exposure, and the possible risks associated to these events in order to support the decision-making process in emergency departments, providing information with regard to different patients in terms of age group or their different injuries as routes of exposure and symptoms.

## 2. Materials and Methods

### Case Series Presentation

**Figure 1:**
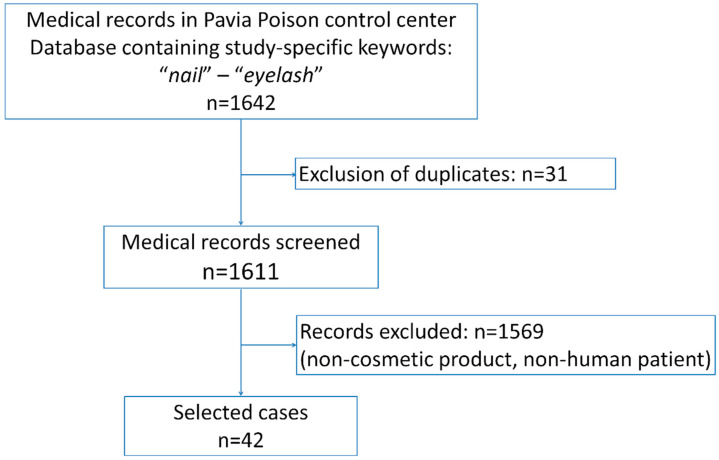
Flow chart of case selection process.

This single centre retrospective study was performed by the toxicology research staff of Pavia Poison Control Centre (PCC)—National Toxicology Information Centre (Pavia, Italy). Most of the consultation’s request came from emergency departments, where the patients were located, from all over the national territory. For each patient, a digitized medical record was compiled, reporting the following: the demographic data, exposure circumstances, agents involved, clinical picture at admission and during hospitalization, laboratory and clinical analyses, adopted treatments, clinical follow-up, and main outcome. In the considered period (January 2007–April 2020), 301,851 medical records were collected from the database. All the consultation requests reporting for nail/false eyelash glue exposure were retrieved using specific keywords. All records involving non-human patients or different products were excluded. Moreover, the use of cyanoacrylate glue not intended for cosmetic use (such as DIY work glue), even if misused as nail/false eyelash glue, represented an exclusion criterion (Figure 1). All cases were anonymized, and the following data were included in the analysis: age, sex, route of exposure, product involved and composition (if known), signs, symptoms, and outcome. Patient groups were defined as follows: Infant: <4 years old, Child: 5–10 years old, Adolescent: 11–17 years old, Adult: >18 years old. In order to identify the intoxication severity, symptoms were classified using the following scale: a score of 0 was used for asymptomatic patients, 1 for patients with mild and spontaneously resolving symptoms (such as first-degree burns and erythema), 2 for patients with severe and more pronounced symptoms (such as second-degree burns or glued eyelid). Data were analyzed descriptively, numerical data were presented as ranges and modes, while nominal data were presented as absolute values and proportions. The study was approved by the local ethics committee (IRB “Istituti Clinici Scientifici Maugeri SpA SB” No.: 2019-2350 CE). This report was prepared following the CARE reporting guidelines [12].

## 3. Results

### 3.1. Patients Demographics, Route of Exposure, and Symptoms

A total of 42 consultation requests due to exposure to nail/false eyelash glue were included. They are summarized in Table 1.

Patients’ ages ranged from 1 to 47 years, with the mode being 2 years old. Thirty patients (71%) were female, while 12 patients were male (29%). Ten patients were completely asymptomatic (score = 0, 24%), while 32 patients presented with symptoms. Among all patients inquiring for medical consultation, the asymptomatic patients came in contact with nail/false eyelash glue via two exposure routes: ingestion (n = 6) or buccal contact (n = 4). Considering symptomatic patients, the routes of exposure were the following: dermal, that is, cutaneous contact (n = 19), buccal contact (n = 5), ocular (n = 5), ingestion (n = 2), and chronic inhalation (n = 1). In seven cases, the contact routes were more than one, as shown in Table 1 (n = 5 dermal-ocular, n = 1 buccal–dermal; n = 1 buccal-ocular). A case of chronic inhalation for occupational exposure has also been included. 

**Figure 2:**
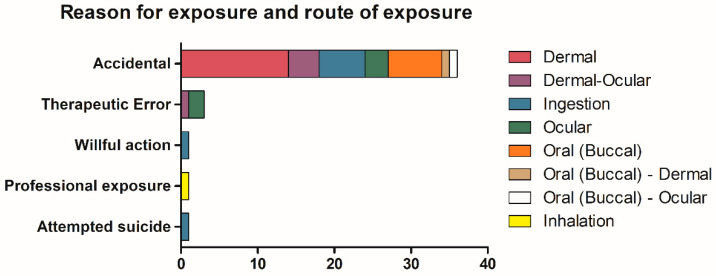
Way of exposure based on route of exposure.

As shown in Figure 2, accidental/unintentional exposure was the most common event (86% of cases, n = 36), mainly due to (i) spillage of the product during the normal procedure of glue application, or (ii) inadvertent child contact, the adhesives being left unattended by adults. In three cases (#18, #31, and #33—Table 1), the exposure was due to a therapeutic error, when the patients (or their tutors) confused the small bottle of nail glue with prescribed eye drops, consequently instilling it directly on the eye. One patient was the victim of wilful actions at school, and the last case was an attempted suicide using co-ingestion of both nail glue and shower gel. In all the above-mentioned cases, an acute single exposure to the agent occurred. Otherwise, chronic exposure to the nail glue was evidenced in the remaining case involving a beautician, who presented with respiratory problems.

**Figure 3:**
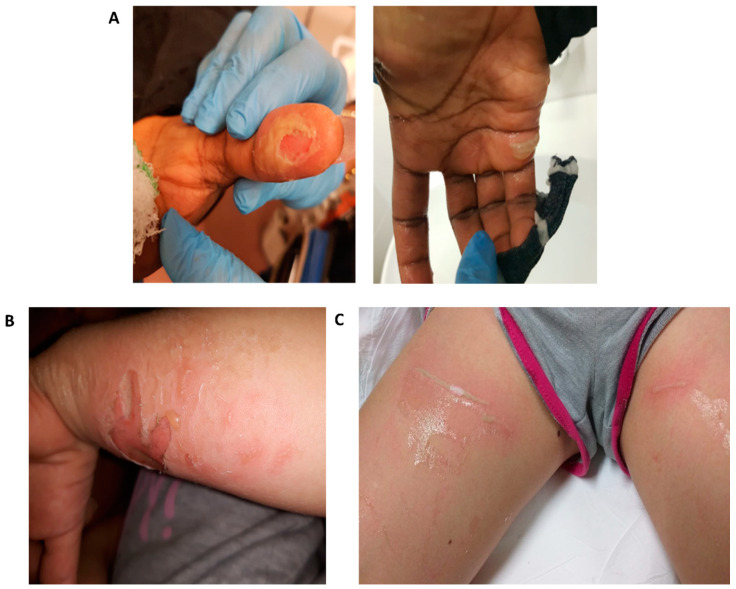
(**A**) Case #11 detail of the thumb and palm of the patient. The panicked patient tried to unglue the thumb with opposite force, consequently tearing the already damaged skin. On the right palm, there is a visible blister. The burn was managed conservatively. (**B**) Case #12 detail of a forearm showing second-degree burns with visible blisters. The burn was managed with surgical wound toilet. (**C**) Case #14 detail of thighs. Blisters and hyperaemia are clearly observable. The burns were treated conservatively.

The cutaneous manifestation after this kind of injury ranges from first- to second-degree chemical burns, hyperaemia, skin damage, and blisters. Particularly, in some cases, the lesions were severe, as shown in Figure 3A–C, respectively.

The different adhesives’ composition was reported in 22 cases (52%) (Table 2). It has to be highlighted that in all symptomatic patients, cyanoacrylate was in the product formulation, with the exception of three cases.

Dermal exposure through cutaneous contact caused symptoms in the majority of patients inquiring for medical consultation (n = 14, 33%); furthermore, the bulk of cases presenting a score of 2 (n = 6 out of 9, 67%) was due to this kind of exposure.

**Figure 4:**
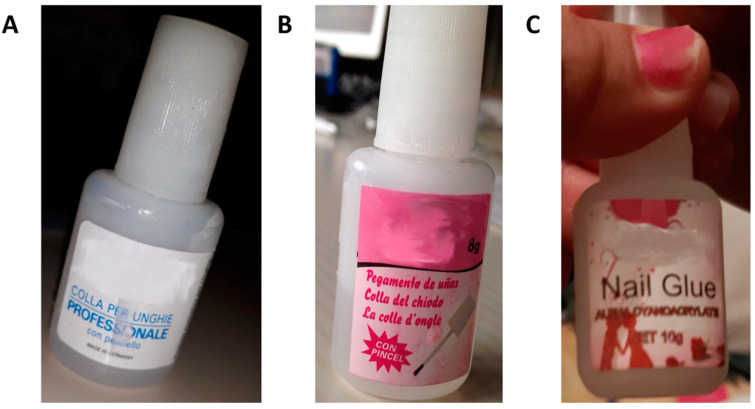
(**A**) Photo of the product involved in case #12. (**B**) Photo of the product involved in case #39. (**C**) Photo of the product involved in case #40. All products have been anonymized.

In Figure 4 are shown some examples of nail glue cosmetic products for which PCC has been consulted.

### 3.2. Treatment Administered to Symptomatic Patients

For all patients, PCC physician toxicologists suggested treating the lesions symptomatically. Cutaneous chemical burns were firstly well-rinsed with saline sterile solution, and then treated with topical antimicrobial unguent (silver sulfadiazine). If needed, the patient underwent surgical wound toilet of the lesion (case #12). If vitrified residues of glue were present, the proposed approach was to gently remove them with petroleum jelly oil. In two/three days, the residue will usually naturally detach from the skin or mucosae. If the exposure was via ingestion or an oral route, through buccal contact, oral gastroprotection (e.g., magnesium/sodium alginate, magaldrate) was prescribed. When exposure occurred via the ocular route, the symptomatic approach became more complicated due to the fragility and sensitivity of the eye tissues. Thus, PCC physicians suggested rinsing the eye for a long time (at least 30 min) with saline sterile solution, trying to remove the glue with a cotton swab soaked with petroleum jelly oil, but not forcing the opening of the eyelids if glued together. If the eyelashes were tightly glued together, the suggested approach was to cut them and verify whether the eyelids could move. In the most severe cases (case #33), when the eyelids were totally glued to the eyeball, an ophthalmic consultation with a specialist was required.

## 4. Discussion

The recent fashionable trend of performing an acrylic nail manicure at home is gaining popularity, and acrylic tips and nail glue (sold separately or in kits) can be easily purchased in cosmetics outlets or online by non-professionally trained individuals. The main component of adhesives is cyanoacrylate, which, when in contact with moisture, produces an exothermic reaction that in the presence of cotton fibres rich in –OH residues (as in jeans or leggings or t-shirts), could make holes in clothes, and even cause them to catch fire [9,13,14,15]. To avoid these accidents, in some (but not all) cyanoacrylate glue safety data sheets, it is suggested to prevent contact with fabric and cellulose (e.g., cotton material) [4,10]. Despite pH possibly being considered as a relevant parameter in this type of injury, it is important to underline that the exothermic reaction produced during the monomers’ polymerization is mainly responsible for these burns [9]. 

In our series, the paediatric population represents the most involved category in the above reported exposures. As cosmetics are not perceived as being hazardous to our health, parents often leave them within children’s reach. Nevertheless, as demonstrated by the reported case study, it is essential to carefully consider the possible occurrence of accidental spillage and dropping of glue as a part of the normal and foreseeable condition of product use. It follows that even beauticians and adult women, when applying or wearing nail tips or false eyelashes, may be considered at risk, both in terms of the documented allergic reaction to these products after long-lasting use or inhalation, as well as for the punctual hazard during the single beauty procedure [10]. Concerning case #28, who was suspected of respiratory problems, the PCC toxicologist suggested that she should visit a specialist at the first consultation (both with an allergist and a lung specialist) to better understand the entity of her self-reported symptoms; unfortunately, during follow-up calls, she informed us that she never had these visits; therefore, no further analysis could be done. The above-reported type of glue polymerizes instantaneously, meaning the therapeutic window is very narrow, and usually, at the time the patients reach the hospital, the only thing physicians can do is to gently remove the vitrified glue. Otherwise, it would be recommended to immediately remove the liquid glue at home and then irrigate the area (even the eye) before calling a PCC or going to the hospital. 

According to previous evidence in the literature [9] and based on presently reported case series and available data, the dermal route of exposure via cutaneous contact is frequently associated with symptoms, with all patients being symptomatic. Even ocular exposure can be dangerous, especially if a therapeutic error occurs when the adhesive is confused with eye drops, as in the cases #18, #31, and #33. In case #33, the clinical feature appeared critical since the consultation request arrival because of the young age of the patient who could not collaborate, and also because the eye and even the eyelashes were tightly glued together. At the first follow-up, the ophthalmic specialist confirmed the impossibility of opening the eyelids. Unfortunately, the patient was moved to a central hospital and all the attempts to follow-up his clinical course failed.

Ingestion led to mild symptoms in only two patients out of eight. More precisely, concerning the symptomatic cases: patient #26, very scared, had pharyngodynia, glottis oedema and hyperaemia, which were evaluated as mild by the physician who visited the woman; patient #27 suffered from epigastralgia but this event was an attempted suicide with co–ingestion of nail glue and shower gel, so it was not possible to discriminate which product induced the symptomatology. Furthermore, it has to be underlined that even in presence of cyanoacrylate, which is the most dangerous component of nail glue, in three cases of ingestion, patients were asymptomatic. Oral exposure through buccal mucosa contact was recorded in seven exposure events. Symptoms appeared evident only in three cases, consisting of solid glue residue attached to the mouth and/or to the teeth, accompanied by mild pharyngodynia. In contrast, the other four patients were asymptomatic, thanks to the timely intervention of the parents who were quick to remove the product and vigorously wash the mouths of the children. Based on these pieces of evidence, ingestion seems to be a less dangerous exposure route, even according to the literature; in fact, Eyth and colleagues reported the cases of two children with no sign of burns after ingestion of glue with only vitrified residues of glue attached to the oral cavity, pharynx and trachea, and bronchi, respectively [9]. Nevertheless, selection bias is extremely probable, due to the less frequent consultation request to PCC in the case of asymptomatic dermal contact, representing this as a limitation of this study. 

Unfortunately, it was not possible to quantify the doses of exposure, since patients often spilled all the contents of the bottle, and they were not able to refer to the original volume of the product nor estimate how much product was spilled. There may be an association with the dose, as also confirmed by the literature [9], but it is impossible to quantify the strength of this association using the available data. Product quantity may be much less in ingestion and buccal contact with respect to spillage and cutaneous exposure.

Based on these data, it could be useful to add safety cap mechanisms to these adhesives to effectively prevent children from opening it [8]. Moreover, almost all commercially available nail glues are packaged in flacons, which are extremely similar in size, shape, and opening mechanism to eye drop bottles, and this can lead to dangerous confusion and therapeutic errors, and it is not unusual that people, in making a mistake, may instil glue into their own eyes [8,16]. 

This problem was first raised in 1984, and later in 1990 and 2001 by other authors highlighting the necessary requirement of changing the packaging of these products, but until today, nothing has been done to consistently solve this problem [17,18,19]. Even the labels of these products appear totally inadequate to explain their potential hazardous properties, including risks of burns, injuries, and lesions. Often nail glue is sold as false eyelash glue or used in its place, and the product labels are cramped and unreadable due to character dimensions and the text often being faded or cancelled out. Even after toxicologists’ requests, it is not always easy to obtain correct information about the product, not even by contacting the manufacturing company. Thus, it is impossible to be confident about the real composition of the adhesive products. In fact, adulterations and/or falsification of labels cannot be excluded, especially for the products coming from countries outside of Europe, usually bought in temporary shops and online outlets and whose origins cannot be well-traced. For example, the product involved in case #4 was sold as “nail glue”, but raised some concerns about its composition; in fact, the first ingredient was methacrylic acid, which is more likely to be the main component of “nail primer” rather than “nail glue” [20].

Symptomatic treatment is the only accessible strategy; no specific antidote or perfect solvent exists, and therefore, petroleum jelly oil results in being the safest treatment. Acetone could also be used, but according to PCC experience, the risk of increasing skin irritation or lesions is too high, especially in the presence of ocular contact [9]. In each case of ingestion managed by the PCC, an endoscopy of the gastrointestinal tract has never been suggested or performed, because patients who ingested or had contact with oral mucosae never presented with gastrointestinal symptoms nor other kinds of severe symptoms (e.g., intensive drooling, pain, vomiting) which could correlate with more extended lesions through the gastrointestinal tract. Moreover, patients are often young children for whom an invasive procedure such as an endoscopy is not viable, unless strictly essential; in rare or borderline cases, a visit with an otorhinolaryngologist could be sufficient to identify any lesions or any vitrified glue residues if present, as also reported in other studies [9]. 

All the presented data were collected using Pavia Poison Control Centre medical records which were written by toxicologists who provide phone consultations to other physicians for patients who are actually located elsewhere in the national territory. This represents a strong limitation for many reasons, firstly because Pavia toxicologists cannot actually visit patients and they have to perform their consultation using telemedicine and lesion images (often faded or blurred), relying on the information that the actual physician of the patient gives to them. Time presents as a strong enemy to precise anamnesis, and emergency department physicians often have to deal with other urgencies and the time they dedicate to the description of a precise anamnesis is too short. Sometimes, what is extremely important for a toxicologist (the information written on the product’s label, or the modality of the accident) may not be relevant to an emergency physician, and even the patient themselves may underestimate the importance of bringing the product with them, or they may omit fundamental details or read the label erroneously. Moreover, for PCC toxicologists, it is often very difficult to keep in contact with patients who have mild symptoms, who are usually discharged on the same day of admission, and for whom follow-ups are hard to carry out, with a high drop-out rate. Further studies may be directed to collect more patients with successful short-term follow-up calls to control the healing process step-by-step. Moreover, it would be interesting to group together the patients with chronic exposure to these products (as beauticians, or frequent false nail bearers) in order to identify possible injuries due to professional/chronic exposure.

## 5. Conclusions

In conclusion, the present manuscript, reporting a high number of different cases in terms of age group, routes, and types of exposure compared to the available literature, has updated, broadened, and reinforced the knowledge on these unusual injuries. It raises awareness even among emergency department physicians about the underestimated risks associated to the exposure to nail/false eyelash glue made of cyanoacrylate, often perceived as harmless, and how to promptly treat these lesions was described.

## Figures and Tables

**Table 1:** Main characteristics of 42 cases of exposure to nail/false eyelash glue, 1 January 2007–30 April 2020.

Case Number	Age	Clinical Effects	Route of Exposure	Way of Exposure ^1^	Score	Product	Component 1	Component 2	Component 3
1	Adult	Damaged skin	Dermal	A	1	Nail glue	Poly methyl methacrylate		
2	Adult	Finger’s oedema	Dermal	A	1	Nail glue	-		
3	Infant	Second-degree burns on hand and thigh	Dermal	A	1	Nail glue	Cyanoacrylate		
4	Adult	First-degree burns on thigh	Dermal	A	1	Nail glue	Methacrylic acid	Butyl acetate	Butyl methacrylate
5	Adult	Cutaneous hyperaemia and erithema on hand	Dermal	A	1	Nail glue	Cyanoacrylate		
6	Adolescent	Cutaneous hyperaemia and blister	Dermal	A	1	Nail glue	Cyanoacrylate	Poly	
7	Child	Damaged skin	Dermal	A	1	Nail glue	Ethyl cyanoacrylate	Poly	
8	Infant	First-degree burns and residues of glue	Dermal	A	1	Nail glue	-		
9	Infant	Second-degree burns on thigh, perilesional oedema	Dermal	A	2	Nail glue	-		
10	Adolescent	Second-degree burns on thigh	Dermal	A	2	Nail glue	-		
11	Adult	First- and second-degree burns on hand	Dermal	A	2	Nail glue	-		
12	Infant	Second-degree burns on forearm, palm and thigh	Dermal	A	2	Nail glue	Cyanoacrylate	Isopropyl alcohol	Ethanol
13	Infant	Second-degree burns on thigh and thorax	Dermal	A	2	Nail glue	-		
14	Adolescent	First- and second-degree burns on both thighs	Dermal	A	2	Nail glue	-		
15	Adolescent	Glued eyelids	Dermal-Ocular	A	1	Nail glue	Cyanoacrylate		
16	Adult	Conjunctival hyperemia, cutaneous erythema	Dermal-Ocular	A	1	Nail glue	-		
17	Adolescent	Glued eyelashes	Dermal-Ocular	A	1	Nail glue	Ethyl cyanoacrylate		
18	Adult	Glued eyelids	Dermal-Ocular	Th. E	1	Nail glue	Cyanoacrylate		
19	Infant	Eyelid and cheek oedema, glued eyelid	Dermal-Ocular	A	2	Nail glue	Cyanoacrylate	Methacrylate	
20	Infant	Asymptomatic	Ingestion	A	0	Nail glue	-		
21	Infant	Asymptomatic	Ingestion	A	0	Nail glue	Cyanoacrylate		
22	Adolescent	Asymptomatic	Ingestion	W	0	Nail glue	Cyanoacrylate		
23	Adult	Asymptomatic	Ingestion	A	0	False eyelashes glue	Cellulose	Dodecyl benzene sulfonate	Ammonium hydroxide
24	Adolescent	Asymptomatic	Ingestion	A	0	Nail glue	-		
25	Infant	Asymptomatic	Ingestion	A	0	Nail glue	Ethyl cyanoacrylate		
26	Adult	Pharyngodynia, glottis oedema, hyperaemia	Ingestion	A	1	Nail glue	-		
27	Adult	Epigastralgia	Ingestion	S	1	Nail glue	-		
28	Adult	Cough and chronic irritation	Inhalation	Pr	1	Nail glue	Cyanoacrylate		
29	Adult	Slight ocular discomfort	Ocular	A	1	Nail glue	Cyanoacrylate		
30	Child	Oculodynia, conjunctival hyperemia	Ocular	A	1	Nail glue	Ethyl cyanoacrylate		
31	Adolescent	Oculodynia	Ocular	Th. E	1	Nail glue	-		
32	Child	Conjunctival hyperemia, oedema and burning eyelids	Ocular	A	1	Tip glue	Ethyl cyanoacrylate	Methyl methacrylate	BHA
33	Infant	Glued eyelid and eyelashes	Ocular	Th. E	2	Nail glue	-		
34	Infant	Asymptomatic	Oral (Buccal)	A	0	Nail glue	-		
35	Infant	Asymptomatic	Oral (Buccal)	A	0	Nail glue	-		
36	Infant	Asymptomatic	Oral (Buccal)	A	0	Nail glue	-		
37	Infant	Asymptomatic	Oral (Buccal)	A	0	Nail glue	-		
38	Infant	Glue attached to palate and teeth	Oral (Buccal)	A	1	Nail glue	Ethyl cyanoacrylate	Poly methyl methacrylate	BHA
39	Adult	Pharyngodynia	Oral (Buccal)	A	1	Nail glue	-		
40	Infant	Solid and dry residues of glue on mouth	Oral (Buccal)	A	1	Nail glue	-		
41	Adult	Cutaneous burns, dysphonia, pharyngodynia	Oral (Buccal)-Dermal	A	2	Nail glue	Ethyl cyanoacrylate		
42	Infant	Glued tongue and eyelids	Oral (Buccal)-Ocular	A	1	Nail glue	Cyanoacrylate		

^1^ Legend for Way of exposure: A: Accidental; Th. E: Therapeutic Error; W: Wilful action; Pr: Professional Exposure; **S**: Attempted suicide

**Table 2:** Glue composition vs. clinical scores.

Glue Composition	PubChem^®^ CID ^1^	Score 0	Score 1	Score 2	Total
Cyanoacrylate	81530	2	7	0	9
90% Cyanoacrylate, 10% Poly	81530–6658	0	1	0	1
Cyanoacrylate, Methacrylate	81530–6658	0	1	0	1
Cyanoacrylate, Isopropyl alcohol, Ethanol	81530–3776–702	0	0	1	1
Ethyl cyanoacrylate	81530	1	2	1	4
Ethyl cyanoacrylate, Poly	81530–6658	0	1	0	1
Ethyl cyanoacrylate, Methyl methacrylate, BHA	81530–6658–24667	0	1	0	1
Ethyl cyanoacrylate, Poly methyl methacrylate, BHA	81530–6658–24667	0	1	0	1
Poly methyl methacrylate	6658	0	1	0	1
Methacrylic acid, Butyl acetate, Butyl methacrylate	4093–31272–7354	0	1	0	1
Cellulose, Dodecyl benzene sulfonate, Ammonium hydroxide	14055602–15900–12896473	1	0	0	1
Unknown composition	-	6	7	7	20
Total					42

^1^ PubChem^®^ CID refers to: https://pubchem.ncbi.nlm.nih.gov/.
